# Development of a hydrolysis probe-based real-time assay for the detection of tropical strains of *Fusarium oxysporum* f. sp. *cubense* race 4

**DOI:** 10.1371/journal.pone.0171767

**Published:** 2017-02-08

**Authors:** Jaime Aguayo, Diane Mostert, Céline Fourrier-Jeandel, Isabelle Cerf-Wendling, Bruno Hostachy, Altus Viljoen, Renaud Ioos

**Affiliations:** 1 ANSES, Laboratoire de la Santé des Végétaux-LSV, Unité de mycologie. Domaine de Pixérécourt, Malzéville, France; 2 Department of Plant Pathology, Faculty of AgriSciences. Stellenbosch University. Matieland, South Africa; 3 ANSES, Laboratoire de la Santé des Végétaux-LSV, Unité des ravageurs et agents pathogènes tropicaux. Saint-Pierre, Ile de la Réunion, France; Bhabha Atomic Research Centre, INDIA

## Abstract

*Fusarium oxysporum* f. sp. *cubense* (Foc) is one of the most important threats to global banana production. Strategies to control the pathogen are lacking, with plant resistance offering the only long-term solution, if sources of resistance are available. Prevention of introduction of Foc into disease-free areas thus remains a key strategy to continue sustainable banana production. In recent years, strains of Foc affecting Cavendish bananas have destroyed plantations in a number of countries in Asia and in the Middle East, and one African country. One vegetative compatibility group (VCG), 01213/16, is considered the major threat to bananas in tropical and subtropical climatic conditions. However, other genetically related VCGs, such as 0121, may potentially jeopardize banana cultures if they were introduced into disease-free areas. To prevent the introduction of these VCGs into disease-free Cavendish banana-growing countries, a real-time PCR test was developed to accurately detect both VCGs. A previously described putative virulence gene was used to develop a specific combination of hydrolysis probe/primers for the detection of tropical Foc race 4 strains. The real-time PCR parameters were optimized by following a statistical approach relying on orthogonal arrays and the Taguchi method in an attempt to enhance sensitivity and ensure high specificity of the assay. This study also assessed critical performance criteria, such as repeatability, reproducibility, robustness, and specificity, with a large including set of 136 *F*. *oxysporum* isolates, including 73 Foc pathogenic strains representing 24 VCGs. The validation data demonstrated that the new assay could be used for regulatory testing applications on banana plant material and can contribute to preventing the introduction and spread of Foc strains affecting Cavendish bananas in the tropics.

## Introduction

Fusarium wilt of banana is caused by the soil-borne fungus *Fusarium oxysporum* f. sp. *cubense* (Foc). The fungus comprises three races, which are described according to the host cultivar that they affect [1–3]. Foc race 4 is considered the most economically important race as it affects Cavendish bananas, which comprise 40% of all bananas grown [4]. It also attacks many other banana varieties, and thus represents a serious risk to food security in countries where this crop is consumed as staple food [5–7].

Foc race 4 can be subdivided into two groups, Foc subtropical race 4 (Foc STR4) and Foc tropical race 4 (Foc TR4), which are phylogenetically distantly related and whose virulence varies under different environmental conditions [8–10]. Foc STR4 attacks Cavendish bananas only in the subtropics, whereas Foc TR4 attacks the same variety both in the tropics and in the subtropics [3]. Foc is also characterized by vegetative compatibility groups (VCGs). VCGs comprise strains that can anastomose to form stable heterokaryons, and in which genetic material can be shared by compatible individuals [1]. VCGs are therefore relevant evolutionary units that are genetically related [1, 11, 12]. To date, 24 Foc VCGs have been described worldwide [1, 13]. Foc STR4 includes VCG 0120, 0122, 0123, 0126, 0129, 01210, 01211, 01215 and 01219, while Foc TR4 is generally described as a single pathogen clone that corresponds only to VCG 01213/16 [1, 14, 15]. However, recent work on phylogenetics has suggested including VCG 0121 in the Foc TR4 group. Indeed, VCG 0121 has proven to be genetically closely related to VCG 01213/16 and was shown to be aggressive to banana in tropical conditions, although its pathogenicity outside Asia remains unknown [1, 14–16]. VCG 01213/16 is highly aggressive and is the principal VCG responsible for the epidemics of Cavendish bananas in the tropics [13]. Since its emergence, VCG 01213/16 has caused serious damage in Southeast Asia and Northern Australia [3], where it remained confined for several years. However, VCG 01213/16 has recently been detected in the Middle East, Pakistan and Mozambique, showing a troublesome expansion to Western Asia and Africa [7, 17, 18]. VCG 0121 may also attack Cavendish bananas under tropical conditions [19–21]. VCG 0121 was the primary Foc strain responsible for losses of Cavendish bananas in Taiwan before VCG 01213/16 was identified on the island.

In order to prevent the spread of Foc strains affecting Cavendish bananas in the tropics to disease-free areas in Asia, Africa, Latin America and the Caribbean, the Food and Agriculture Organization (FAO) and banana research centers have urged countries to set up global actions against Foc TR4 [22, 23]. Control of Foc TR4, including complete eradication of infected plants and quarantine of infested areas to reduce pathogen dissemination, is often ineffective and has not yet been successful [4, 10, 24]. There is also no immune variety available to replace Cavendish bananas for export and domestic trade. Prevention of introduction is therefore of the utmost importance to control the disease. Consequently, rapid and reliable *in planta* detection methods need to be developed to promptly confirm the identity of Foc TR4 once Cavendish banana plants with suspect symptoms are found, with a view to considering eradication of the disease [6]. Considering that VCG 0121 is genetically close to VCG 01213/16 and that it has the ability to infect Cavendish under tropical conditions, the introduction of strains of any of these VCGs into Cavendish-growing countries in the tropics could result in devastating consequences for the banana industry.

As a basic requirement for regulatory analysis, detection methods must provide repeatable and reliable results and must be easily transferable between laboratories. Therefore, optimization of the test and assessment of different performance criteria are necessary to determine potential limitations of the detection technique. Assurance about the reliability of the test will prevent the risk of false negative or false positive results [25], which is particularly crucial with a potentially devastating pathogen such as Foc TR4.

Several molecular markers targeting Foc race 4 [26–30] and VCG 01213/16 [10, 13, 31, 32] have been developed. These techniques made use of conventional and real-time PCR (qPCR), or real-time fluorescent loop-mediated isothermal amplification (LAMP). However, to our knowledge, an optimized and validated identification tool that can simultaneously detect both 01213/16 and 0121, VCGs that cause Fusarium wilt of Cavendish bananas in the tropics, is not yet available. Such a tool would be extremely helpful to prevent the introduction or spread of these destructive pathogens into regions where the Cavendish banana industry is vital, such as Latin America and the Caribbean. Compared to conventional PCR, qPCR offers better performances in terms of sensitivity, specificity and rapidity, which are vital for the detection of quarantine plant pathogens. qPCR also permits quantification of the target DNA [33–36]. LAMP is mostly used as first-instance screening because it can be performed in the field and does not need special thermal cycling equipment. However, it may be less sensitive compared to qPCR, and positive samples can be missed due to the poor quality of the DNA extracts [37].

In this study, we developed a Foc TR4-specific real-time PCR test that targets both VCGs 01213/16 and 0121, and that was optimized to detect the fungi in Cavendish plant material. The test is based on a genetic locus probably associated with virulence [8]. Virulence genes are considered to be ideal targets for molecular identification of *formae speciales* of *F*. *oxysporum* [38–40]. Since there is a strong link between this group of genes and pathogenicity, they have been considered excellent markers for host-specific strains of Foc [8]. Virulence genes made it possible to distinguish strains of different *formae speciales*, different races of the same *forma specialis*, and non-pathogenic forms of *F*. *oxysporum* [1, 41–44].

Using orthogonal arrays (OAs) and the Taguchi method, we optimized the following conditions and parameters of the test: concentration of primers/hydrolysis probe and MgCl_2_, the annealing temperature and the denaturation/polymerization pattern. OAs represent experimental design that helps to minimize resources and turnaround time to determine optimal conditions, and that is widely used in innovative experimentation or product/process improvement projects [45]. The design involves the performance of a series of experiments containing the levels of each tested factor in an equal number of times across the entire array. Coupling OAs and the Taguchi method makes it possible to identify parameters that have significant effects on the efficiency and the sensitivity of the PCR reaction. The validation of the performance criteria of the qPCR test for routine analysis included sensitivity, specificity, repeatability, reproducibility and robustness. In order to evaluate its efficacy and reliability, the assay was tested on artificially and naturally infected banana samples.

## Material and methods

### Fungal collection and DNA extraction

A total of 136 isolates were included in this work (S1 Table). The collection included 73 Foc pathogenic isolates representing 24 VCGs, and 13 Foc non-pathogenic isolates from different origins and banana cultivar hosts. Furthermore, 24 *F*. *oxysporum* isolates corresponding to 16 other *formae speciales*, 18 putative non-pathogenic *F*. *oxysporum*, and eight isolates from seven other *Fusarium* species were included in the study. Foc TR4 and STR4 isolates were maintained in level 3 biohazard containment facilities, in compliance with EU Directive 2008/61/EC.

For DNA extractions, the isolates were first cultured on potato dextrose agar (PDA) medium (difco™) for approximately 5 days, after which 100 to 200 mg of fresh mycelium were harvested using a sterilized scalpel blade. Genomic DNA (gDNA) was extracted using the DNeasy plant mini kit™ (Qiagen, Courtaboeuf, France) following the manufacturer’s guidelines. gDNA concentration was estimated using the Nanodrop 2000 Spectrophotometer™ (Thermo Scientific, Wilmington, DE, USA). The gDNA concentration for all the isolates was adjusted to 0.5 ng kkL^-1^ for further tests. The universal primer pair ITS1/ITS4 [46] (Table 1) was used to check the quality of the DNA extracted and to screen out false negative results caused by potential PCR inhibition, DNA degradation or loss during extraction. PCR conditions were those proposed by the authors [46].

**Table 1:** Characteristics of primers (F/R) and probes (P) used in this study.

Target species	Foc race	VCG	Primer or probe	Sequence (5'- 3')	Target gene
*F*. *oxysporum*	Tropical	01213/16,	FWB-TR4 F^a^	CGGTCTCGGCCAAATCTGATT	Gene coding for a hypothetical protein
f. sp *cubense*	race 4	0121	FWB-TR4 R	ACGACTTATCTAGCGGTTGATGTG	
			FWB-TR4 P	ACCCTTCAACTCCACTCGATCGCA	
*F*. *oxysporum*	Tropical	01213/16,	0422 F2^b^	GGCTTCCAGACCGACAAGATAT	SCAR
f. sp *cubense*	race 4	0121	0422 R2	TGCTTGGCCTTGATTCTGACT	
			0422 P2	ATAATCGAACAGTTTGCG	
*F*. *oxysporum*	Tropical	01213/16	W2987 F^c^	TGCCGAGAACCACTGACAA	Gene coding for a hypothetical protein
f. sp *cubense*	race 4		W2987 R	GCCGATGTCTTCGTCAGGTA	
*F*. *oxysporum*	Tropical	01213/16	Foc TR4 F^d^	CACGTTTAAGGTGCCATGAGAG	IGS
f. sp *cubense*	race 4		Foc TR4 R	CGCACGCCAGGACTGCCTCGTGA	
*F*. *oxysporum*	Tropical	01213/16	VCG01213/16 F1^e^	ACGTTTAAGGTGCCATGAGAG	IGS
f. sp *cubense*	race 4		VCG01213/16 R2	CCTCGTGAGCCACTTTTTAT	
*F*. *oxysporum*	Race 4	01213/16, 0121, 0120, 0122,	Foc-1^f^	CAGGGGATGTATGAGGAGGCT	RAPD
f. sp *cubense*		0123, 0126, 0129, 01210,	Foc-2	GTGACAGCGTCGTCTAGTTCC	
		01211, 01215, 01219			
Fusarium strains containing the putative pathogenicity gene	-	-	356 F^c^	GAGTATGCYGATATGGTRTTG	Gene coding for a hypothetical protein
			357 R	CACAGCGCATGTTGATGAAC	
Plant or fungus	-	-	18S uni F^g^	GCAAGGCTGAAACTTAAAGGAA	ITS
universal primer			18S uni R	CCACCACCCATAGAATCAAGA	
			18S uni P	ACGGAAGGGCACCACCAGGAGT	
Fungus universal	-	-	ITS1^h^	CTTGGTCATTTAGAGGAAGTAA	ITS
primer			ITS4	TCCTCCGCTTATTGATATGC	

References

^a ^This study

^b^[29]

^c^ [8]

^d^ [10]

^e^ [13]

^f^ [26]

^g^ [47]

^h^[46].

### Design of real-time PCR primers and hydrolysis probe

In their work on the genomics of Foc, Li et al. [8] described a genetic locus from mutant W2987 expected to be associated with Foc TR4 virulence. Based on *in vitro* pathogenicity tests, Li et al. [8] suggested that the target gene may be involved in the pathogenesis of Foc TR4, although further characterization by gene deletion and complementation was still needed to confirm its function. The authors did not name the gene and they refer to it as a “putative virulence gene” that encodes a hypothetical protein with unknown function. The present work took this gene as a basis for the development of the test.

A selected panel of 34 isolates was sequenced in this study using the primer set 356F/357R (see S1 Table for GenBank accessions), which was designed to specifically amplify this locus (Table 1), following the PCR conditions recommended by Li et al. [8]. Sequences were analyzed together with those published by Li et al. [8] in GenBank (accession numbers JX090599-JX090603) using the Muscle aligning tool [48] implemented in Geneious R6 (Biomatters, Auckland, New Zealand). Primer 3 [49], available in Geneious R6, was used to manually design three pairs of Foc TR4-specific forward and reverse primers, as well as hydrolysis probes, which were evaluated for their respective thermodynamic characteristics and potential secondary structures. A first evaluation of the analytical specificity (i.e. the ability of the test to identify target from non-target) of the three candidate primer pairs and probes was performed *in silico* using the primer blast algorithm [50] and the Broad FGI *Fusarium* Comparative Database (http://www.broad.mit.edu/annotation/genome/fusarium_group/MultiHome.html).

A preliminary *in vitro* evaluation of the three candidate primers was performed on a subset of 40 isolates (Table 2) by qPCR using a sybr-green intercalating dye. Tests were performed using the Mesa Green qPCR™ Master mix Plus for sybr® assay (Eurogentec) on 20 μL reactions mixtures containing 1X reaction buffer, 0.2 μM of R/F primers, 2 μL of DNA template and molecular-grade water (MGW). PCR cycling conditions included an initial denaturation step at 95°C for 10 min, followed by 40 cycles of a 10 s denaturation step at 95°C and a hybridization/polymerization step at 60°C for 45 s. Analysis of the melting curves, the candidate primer specificity and their proneness to form secondary structures, permitted the selection of the FWB-TR4 F/R primer pair. Further tests were performed in combination with the hydrolysis probe, FWB-TR4 P. Hereafter the combination of the forward and reverse primers and the probe will be referred to as FWB-TR4, which stands for Fusarium wilt of banana TR4.

**Table 2:** Response of *Fusarium* strains to some existing *Fusarium oxysporum* f. sp. *cubense* detection tools.

Isolate	Species	Forma speciale	VCG	Foc1-Foc2^a^	W2987F/R^b^	FocTR4F/R^c^	01213/16F1/R2^d^	Foc4-0422F2/R2/P2^e^	FWB-TR4 F/R/P^f^
CAV180	*F*. *oxysporum*	*cubense*	0121	+	-	-	-	+	+
CAV195	*F*. *oxysporum*	*cubense*	01219	+	-	-	-	-	-
CAV1100	*F*. *oxysporum*	*cubense*	0129	+	-	-	-	-	-
CAV189	*F*. *oxysporum*	*cubense*	01214	-	-	-	-	-	-
CAV293	*F*. *oxysporum*	*cubense*	0120	+	-	-	-	-	-
CAV312	*F*. *oxysporum*	*cubense*	01213/16	+	+	+	+	+	+
CAV613	*F*. *oxysporum*	*cubense*	0126	+	-	-	-	-	-
CAV786	*F*. *oxysporum*	*cubense*	0124	-	-	-	-	-	-
CAV810	*F*. *oxysporum*	*cubense*	01213	+	+	+	+	+	+
CAV929	*F*. *oxysporum*	*cubense*	0123	-	-	-	-	-	-
CAV604	*F*. *oxysporum*	*cubense*	01216	+	+	+	+	+	+
LSVM 1074	*F*. *oxysporum*	*cubense*	----	+	-	-	-	-	-
LSVM 1075	*F*. *oxysporum*	*cubense*	----	+	-	-	-	-	-
NRRL 36118	*F*. *oxysporum*	*cubense*	01221	-	-	-	-	-	-
NRRL 36112	*F*. *oxysporum*	*cubense*	01215	+	-	-	-	-	-
NRRL 36113	*F*. *oxysporum*	*cubense*	01214	-	-	-	-	-	-
NRRL 36115	*F*. *oxysporum*	*cubense*	01224	-	-	-	-	-	-
NRRL 36116	*F*. *oxysporum*	*cubense*	01223	-	-	-	-	-	-
NRRL 36117	*F*. *oxysporum*	*cubense*	01222	-	-	-	-	-	-
NRRL 26029	*F*. *oxysporum*	*cubense*	01210	+	-	-	-	-	-
LSVM 419	*F*. *oxysporum*	*canariensis*	----	+	-	-	-	-	-
LSVM 1024	*F*. *oxysporum*	----	----	-	-	-	-	-	-
LSVM 1025	*F*. *oxysporum*	----	----	-	-	-	-	-	-
LSVM 1026	*F*. *oxysporum*	----	----	-	-	-	-	-	-
LSVM 1027	*F*. *oxysporum*	----	----	-	-	-	-	-	-
LSVM 1028	*F*. *oxysporum*	----	----	-	-	-	-	-	-
LSVM 1029	*F*. *oxysporum*	----	----	-	-	-	-	-	-
LSVM 1030	*F*. *oxysporum*	----	----	-	-	-	-	-	-
LSVM 1031	*F*. *oxysporum*	----	----	-	-	-	-	-	-
LSVM 1032	*F*. *oxysporum*	----	----	-	-	-	-	-	-
LSV M 942	*F*. *oxysporum*	----	----	-	-	-	-	-	-
LSV M 1079	*F*. *oxysporum*	----	----	-	-	-	-	-	-
LSV M 1082	*F*. *oxysporum*	----	----	-	-	-	-	-	-
NRRL13566	*F*. *fujikuroi*	----	----	-	-	-	-	-	-
LSVM 895	*F*. *ananatum*	----	----	-	-	-	-	-	-
LSVM287	*F*. *concentricum*	----	----	-	-	-	-	-	-
NRRL 22402	*F*. *solani*	----	----	-	-	-	-	-	-
LSVM 878	*F*. *poae*	----	----	-	-	-	-	-	-
LSVM286	*F*. *proliferatum*	----	----	-	-	-	-	-	-
LSVM 1018	*F*. *incarnatum*	----	----	-	-	-	-	-	-

References

^a^ [26]

^b^ [8]

^c^ [10]

^d^ [13]

^e ^[29]

^f ^This study

### Optimization of real-time PCR conditions

The PCR product amplified with gDNA from isolate CAV604 (VCG01213/16) using the FWB-TR4 F/R primer pair was inserted into a pcr4-topo plasmid (Invitrogen), following the manufacturer’s instructions. Transformed bacterial cells were cultured overnight at 37°C and then purified using the Nucleospin Plasmid Kit (Macherey-Nagel). Molecular weight and plasmid number of copies were determined and then adjusted to a concentration of 0.1 ng μL^-1^. A calibrated ten-fold serial dilution ranging from two to 24 x 10^5^ plasmid copies/μL (pc μL^-1^) was prepared in MGW. qPCR conditions were optimized to improve sensitivity (i.e. minimize the Ct value for positive samples) without compromising specificity. All reactions were performed using the Eurogentec qPCR Core kit NO ROX. PCR reaction mixtures consisted of 20-μL reaction volumes containing 1X reaction buffer, 4X 0.2 mM dNTPs, 0.5 units of Hot Goldstar Taq polymerase and 2 μL of DNA template. Different concentrations of primers/hydrolysis probes (0.1, 0.2 and 0.3 μM) and MgCl_2_ (4.0, 5.0 and 6.0 mM) were tested in order to optimize the sensitivity and efficiency of the PCR reaction. qPCR cycling conditions consisted of an initial denaturation step at 95°C for 10 min, followed by 40 cycles with a denaturation temperature step at 95°C for 10 s, and different annealing temperatures (60, 62 or 64°C) and denaturation/polymerization time patterns (10/45, 15/45 or 15/30 s s^-1^). Tests were performed on a Rotor-Gene 6500 thermal cycler (Corbett Research, Sydney, Australia) set with an auto gain optimization for each channel performed before the first fluorescence acquisition. Mean cycle threshold (Ct) values were computed using the Rotor-Gene software 1.7.75, setting the threshold line at 0.02.

The experiments were designed following a balanced orthogonal array of 18 experimental combinations (Table 3). The trials were designed in such a way as to ensure that each individual factor was present at least on six settings. Each run was conducted with three different plasmid concentrations (2.4 x 10^1^, 2.4 x 10^2^ and 2.4 x 10^3^ pc μL^-1^) as template, and tested in triplicate. Six non-template controls (NTC) were included in each run in order to check the absence of DNA contamination. The Taguchi method was used to minimize Ct values, under the assumption that the lowest Ct values increase analytical sensitivity. The Taguchi method may be used to accurately predict the factor levels for optimum performance. The method uses quadratic loss functions (signal-to-noise ratios) that penalize deviations from prediction values [51]. In the present study, the signal-to-noise ratio used was the “smaller-the-better” (*n*), described by the function $n=-10{log}_{10}\;\left[\frac{1}{r}\;(\sum_{i=1}^{r}y^{2})\right]$, where *n* = signal-to-noise ratio, *r* = number of repeats and y = Ct value (response variable). The signal-to-noise ratio equation should result in negative *n* values, with values close to zero indicating better conditions. The signal-to-noise ratio, *n*, was analyzed under the following linear model:

n=β0+β1MgCl2+β2primerF+β3primerR+β4probe+β5annealing+β6DP

where *β*_0_ was the intercept; *β*_1_ *MgCl*_*2*_, *β*_2_ *primerF*, *β*_3_ *primerR* and *β*_4_ *probe* were the tested concentrations of MgCl_2_, primer F, primer R and probe on the PCR mix, respectively; *β*_5_ *annealing* the annealing temperatures; and *β*_6_ *D/P* the denaturation/polymerization pattern and their respective coefficients. Linear model assumptions were verified using the R package “car” [52]. The model was fitted separately for each of the three plasmid concentrations. Tukey-Kramer tests were performed to compare differences within the tested levels of the parameters.

**Table 3:** Orthogonal arrays of the six variables and three levels used to optimize PCR conditions for the real-time detection of tropical strains of *Fusarium oxysporum* f. sp. *cubense* race 4. D/P stands for denaturation/polymerization pattern.

Runnumber	MgCl(mM)	Primer F(μM)	Primer R(μM)	Probe(μM)	AnnealingTemp (°C)	D/Ppattern
**1**	4	0.1	0.2	0.2	64	10/45
**2**	6	0.1	0.2	0.3	60	15/45
**3**	5	0.3	0.2	0.1	60	10/45
**4**	6	0.2	0.1	0.2	60	10/45
**5**	4	0.3	0.3	0.2	60	15/45
**6**	4	0.1	0.1	0.1	60	15/30
**7**	6	0.3	0.1	0.1	62	15/45
**8**	5	0.2	0.3	0.3	60	15/30
**9**	5	0.1	0.3	0.1	64	15/45
**10**	4	0.2	0.3	0.1	62	10/45
**11**	6	0.2	0.2	0.1	64	15/30
**12**	4	0.2	0.1	0.3	64	15/45
**13**	5	0.2	0.2	0.2	62	15/45
**14**	5	0.1	0.1	0.3	62	10/45
**15**	6	0.1	0.3	0.2	62	15/30
**16**	4	0.3	0.2	0.3	62	15/30
**17**	5	0.3	0.1	0.2	64	15/30
**18**	6	0.3	0.3	0.3	64	10/45

### Performance values

Performance of the test was evaluated according to the European and Mediterranean Plant Protection Organization (EPPO) standard PM7/98 [53]. According to EPPO, a test is considered as fully validated when it provides data for the following performance criteria: analytical specificity, analytical sensitivity, reproducibility and repeatability. The robustness, i.e. ability of the test to remain unaffected by small but deliberate variations of some reaction parameters, was also included in the validation procedure. All validation and performance experiments were carried out in duplicate for each DNA sample, unless indicated otherwise. Six NTCs were systematically included at different steps to check the absence of contamination in all reactions of conventional or real-time PCR.

Analytical specificity of the FWB-TR4 test was verified with gDNA extracts from the full *Fusarium* collection of 136 strains (S1 Table). The FWB-TR4 test was also compared to other tests currently available in the literature, such as Foc4 0422F2/R2/P2, a hydrolysis probe-based test [29], and Foc1-2, a sybr-green chemistry-based test [26], both described as Foc race 4 detection tools, and conventional PCR tests W2987 F/R [8], VCG01213/16 F1/R2 [13] and Foc TR4 F/R [10]. All of these are specific to VCG 01213/16 (Table 1). Test comparisons were performed on the subset of 40 isolates (Table 2) using gDNA previously adjusted to 0.5 ng μL^-1^. All tests were performed following the prescribed conditions, but using either the qPCR Core kit NO ROX (Eurogentec) or the Mesa Green qPCR™ Master mix Plus for sybr®, for real-time PCR, and the HGS Diamond Taq kit (Eurogentec), for conventional PCR.

Analytical sensitivity (also referred to as the limit of detection) was studied using three methods. First, 16 replicates of three concentrations coming from the plasmid DNA serial dilution (from 2 to 2.4 x 10^5^ pc μL^-1^) were tested during the same PCR run. The lowest concentration of target DNA, that generated at least 90% positive results, was considered the limit of detection of the test. Second, a calibrated ten-fold serial dilution was prepared from plasmid DNA (from 2 to 2.4 x 10^5^ pc μL^-1^) diluted in a background of dry Cavendish banana DNA (banana DNA extraction as described on the following section). Both plasmid DNA serial dilutions (in MGW and in a background of dry Cavendish banana DNA) were tested on the same PCR run and Ct values were compared by a Student’s t-test. An analysis of covariance (ancova) was performed to compare the regressions lines of the generated standard curves, with background matrix as the categorical factor with two levels (banana DNA or MGW), the estimated log concentration of the plasmid copies as the regressor and the Ct as the response variable. Finally, the analytical sensitivity between tests was assessed from a calibrated ten-fold serial dilution of gDNA from the Foc TR4 isolate CAV604 ranging from 0.5 ng μL^-1^ to 1 x 10^−4^ ng μL^-1^, comparing the same tests described above for analytical sensitivity.

Repeatability, reproducibility and robustness were evaluated with plasmid DNA controls from CAV604 (VCG 01213/16) containing 2.4 x 10^2^, 2.4 x 10^3^ and 2.4 x 10^5^ pc μL^-1^. gDNA from isolates CAV1100 (VCG 0129), CAV786 (VCG 0124) and CAV613 (VCG 0126) were also included as specificity negative controls. Repeatability was evaluated with ten replicates of the same DNA template in the same run, while reproducibility was assessed evaluating one replicate of the same DNA templates in different runs. Inter-and-intra-coefficients of variation (CV %) based on Ct values were computed for repeatability and reproducibility, respectively. Robustness was evaluated by a variation of ±10% of the optimized reaction volume and the DNA template. The optimized hybridization temperature was also modified by ±2°C. To evaluate the transferability of the test, the assay was performed on another real-time platform, i.e. a LightCycler 480 Real-Time PCR system (Roche), and using the Eurogentec MasterMix NO ROX with the optimized conditions. Normality distribution of the data was verified by the Shapiro-Wilk test and by Q-Q plots. Data were analyzed either by Student’s t-test or a Mann-Whitney-Wilcoxon test to check for differences in the parameters.

### Inoculation assay for specific in planta detection

Colonies of isolates CAV810 (VCG 01213/16), CAV1100 (VCG 0129), CAV613 (VCG 0126) and CAV189 (VCG 01214) were cultured for 15 days at 22°C on PDA media. Approximately 20–25 plugs taken from the margins of actively growing cultures were used to inoculate four 1000-mL Duran® graduated laboratory bottles containing approximately 500 g of autoclaved millet seeds and liquid PDA media. One extra control bottle was prepared containing only millet seeds and liquid PDA media. Inoculated millet seeds were incubated for 1 month at 22°C, and bottles were shaken manually every 2 days. Individual amounts of samples were then prepared by filling 50-mL conical Eppendorf® tubes with the inoculated and non-inoculated (control) millet seeds.

Nineteen Cavendish banana plants were inoculated with the VCG 01213/16 samples, and four Cavendish banana plants were each inoculated with the other VCGs and the non-inoculated control samples. The millet seeds were added onto the potting soil close to the stem of each plant. The plants were placed in an S3-type greenhouse with environmental conditions set at 26°C, 80% humidity and a 12 h light cycle. After 40 days, the plants were removed from their pots and their roots were rinsed with sterile water and 70% ethanol, until all debris or adhering soil was removed. Plants were subsequently cut in half using a sterilized scalpel. From one half, vascular strands were dissected and placed on sterile filter paper in order to dry the tissue (Pérez-Vicente et al., 2014). After 24 hrs., three 20-mg dried tissue subsamples from each plant were each transferred to an individual sterile Lysing Matrix A tube (MP Biomedicals) and kept at room temperature until DNA extraction. From the other half, vascular strands were dissected (100 mg wet weight) and were placed directly in individual Lysing Matrix A tubes, and stored at -20°C until DNA extraction.

The qiagen® dneasy plant mini kit (Cortaboeuf, France) was used for DNA extraction of both types of samples. For each Lysing Matrix A tube, 400 μL of AP1 buffer and 4 μL of RNase A (both supplied with the dneasy plant mini kit) were added. Samples were then disrupted by shaking for two periods of 60 s using the FastPrep®-24 system (MP Biomedicals) with a velocity setting of 6 m s^-1^. DNA extraction was then performed according to the manufacturer’s guidelines, with a final elution containing 100 μL of DNA. DNA was quantified using the Nanodrop 2000 Spectrophotometer (Thermo Scientific). Each DNA sample was subsequently diluted 10 times (1/10^th^) or 100 times (1/100^th^) in AE elution buffer provided with the DNA extraction kit. An anova was performed to test the effect of the type of sample (dry or frozen tissue), the DNA dilution (1/10^th^ or 1/100^th^ dilution) and the interaction (type of sample and DNA dilution) with Ct values as the response variable. Post hoc pairwise Tukey HSD tests were conducted to detect significant mean differences.

In order to assess the presence of PCR inhibitory components, raw DNA extracts, 1/10^th^ and 1/100^th^ dilutions of all the Foc TR4 positive samples were tested in PCR reactions using the 18S uni-F/R/P universal primers/probe test. Comparison of the 18S uni Ct values yielded with dry and frozen samples was performed using a Mann-Whitney-Wilcoxon rank sum test. Average, minimum and maximum 18S uni Ct and DNA concentrations for positive samples were computed to estimate a range of putative values for which a DNA extract was deemed suitable for PCR. Theoretical diluted DNA concentrations for the 1/10^th^ or 1/100^th^ dilutions were computed using the DNA concentrations estimated with the Nanodrop 2000 Spectrophotometer as a reference.

### Tests on field-collected banana samples

Banana tissue collected from plants with Fusarium wilt symptoms was used to assess the performance of the tests with field samples. The sample set included 11 symptomatic banana stem samples from Mozambique, three banana samples from Trinidad and Tobago, and four samples from Reunion Island. Both Trinidad and Tobago and Reunion Island are Foc TR4-free zones (S2 Table). DNA extraction was performed from the dried banana tissues with the qiagen® dneasy plant mini kit, as explained previously. Two banana leaves from the Reunion Island showing symptoms of *Pseudocercospora* spp. were also included. DNA from these samples was extracted following the protocol from Arzanlou et al. [54]. The quality of the DNA was checked using the 18S uni-F/R/P universal primer pair test, as described by Ioos et al. [47].

## Results

### Design and preliminary evaluation of real time PCR primers and probe

Thirty-four new sequences of the putative virulence gene were obtained during this study. All the sequences were deposited in GenBank with accessions numbers KX463694–KX463727 (S1 Table). Alignment of sequences allowed the design of the primers/probe in the FWB-TR4 test (Table 1) to amplify a 180-bp region when used in PCR with target DNA. The *in silico* analytical specificity of FWB-TR4 could only be confirmed for VCG 01213/16 because sequences from VCG 0121 for this gene were absent from GenBank. The DNA regions targeted by the FWB-TR4 primers/probe were all 100% conserved within all the VCG 01213/16 isolates included in this work. However, the TR4-R primer shared 100% identity with the orthologous region in *F*. *oxysporum* f. sp. *raphani* of the putative virulence gene (strain PHW815) and *F*. *oxysporum* f. sp. *radicis*-*lycopersici* (strain CL57). Preliminary *in vitro* analysis with Sybr-Green intercalating dye confirmed the specificity for VCGs 01213/16 and 0121, and the absence of secondary structures of the FWB-TR4 F/R primers. Further tests were performed in combination with the FWB-TR4 P-specific probe.

### Optimization of real-time PCR conditions

Results of the optimized process for the three plasmid concentrations (2.4 x 10^1^, 2.4 x 10^2^ and 2.4 x 10^3^ pc μL^-1^) are summarized in S3 Table. All three plasmid concentrations presented significant effects on the signal-to-noise ratio (*n*) for the probe concentration, the annealing temperature and the denaturation/polymerization pattern. Lower *n* values were obtained for a 0.1 μM probe concentration and a D/P pattern of 10/45 s s^-1^. Although the lowest *n* value for the annealing temperature was obtained with 64°C, further specificity tests on gDNA showed some late non-specific amplification on non-target Foc races at 64°C and 62°C. Therefore, the annealing temperature was adjusted and set up at 60°C for further tests. The MgCl_2_ concentration had a significant effect on the 2.4 x 10^2^ and 2.4 x 10^3^ pc μL^-1^ concentrations. Despite this significant effect, no statistical differences were observed between the tested MgCl_2_ concentrations. The MgCl_2_ concentration was set at 5 mM. Concentrations of the TR4-F/R primers did not present any statistical effect on any of the plasmid concentrations, and their concentration was kept at 0.1 μM. The optimized mix for routine analysis resulted in 20 μL total reaction volumes using the qPCR Core kit NO ROX (Eurogentec) containing MGW, 1X reaction buffer, 5 mM MgCl_2_, 4 x 0.2 mM dNTPs, 0.3 μM TR4-F/R primers, 0.1 μM TR4-P hydrolysis probe, 0.5 U of Hot Goldstar Taq polymerase and 2 μL of template DNA. qPCR optimized conditions were settled at 95°C for 10 m for the initial denaturation step, followed by 40 cycles of denaturation at 95°C for 10 s and annealing at 60°C for 45 s.

### Performance criteria

The FWB-TR4 qPCR test using a hybridization/polymerization temperature set at 60°C yielded positive results with DNA from VCGs 01213/16 and 0121 regardless of the geographical origin and the banana variety, confirming the specificity of the test under these conditions. No cross amplification was detected with DNA from any other Foc VCGs, *formae speciales* of *F*. *oxysporum* or other *Fusarium* species tested (S1 Table).

The analytical specificity of existing conventional and real-time PCR tests was also assessed with the subset of 40 isolates (Table 2). All conventional PCR tests targeting Foc TR4 were exclusively specific to VCG 01213/16. The Foc4 0422 F2/R2/P2 hydrolysis probe-based test, described as a Foc race 4 detection tool, yielded positive results with DNA from both VCGs 01213/16 and 0121, but not from Foc STR4-described VCGs. The Sybr-Green chemistry-based Foc1-Foc2 test yielded positive results for all Foc TR4 and Foc STR4 VCGs, but cross-reacted with two strains of Foc race 1 and with a strain of *F*. *oxysporum* f. sp. *canariensis*, and was therefore not kept for further evaluations (Table 2).

**Fig 1:**
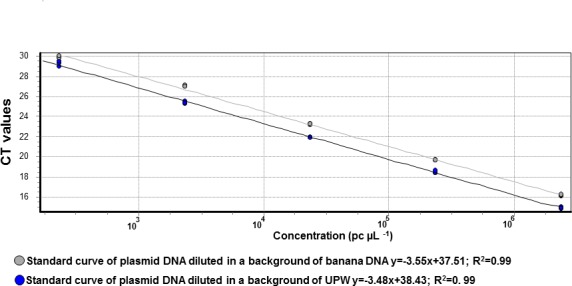
FWB-TR4 qPCR standard curve built with a 10-fold serial dilution of plasmid DNA positive control diluted in water (grey line) or in a background of banana DNA (black line).

Concerning sensitivity, the FWB-TR4 test successfully yielded 94% repeatable positive results with as few as 24 plasmid copies of target DNA per reaction tube. Comparison of standard curve slopes from plasmid DNA diluted either in MGW or in a background of banana DNA by ancova did not show significant interaction between the plasmid log concentration and the background matrix (*p* = 0.29). No significant differences in the Ct values were observed (*p* = 0.524), which suggested that both regression slopes were similar (Fig 1). This means that although some differences were found in the magnitude of the Ct values (mean Ct difference ± SD of 1.2±0.27), with a slight loss of sensitivity for the plasmid DNA diluted on dry banana DNA background, the observed differences were not significant and that no differences were found on the rate of change. Comparisons between tests for analytical sensitivity showed that the two qPCR hydrolysis probe-based tests (FWB-TR4 and Foc 0422F2/R2/P2) were similar (1 x 10^−3^ ng μL^-1^), and that both showed higher sensitivity when compared to conventional PCR assays (Table 4).

**Table 4:** Sensitivity comparison between five detection tools used for *F*. *oxysporum* f. sp. *cubens*e. Tests were performed on three replicates. A positive amplification is represented by +, while a negative result is represented by–for each replicate. Parentheses show a weak positive amplification in conventional PCR.

	Genomic Foc TR4 target DNA (ng/μL)			
Test	1	1x10^-1^	1x10^-2^	1x10^-3^
FWB-TR4-F/R/P^a^	**+++**	**+++**	**+++**	**+++**
Foc4 0422F2/R2/P2^b^	**+++**	**+++**	**+++**	**+++**
W2987 F/R^c^	**+++**	**+++**	**---**	**---**
VCG01213/16 F1/R2^d^	**+++**	**+++**	**+++**	**---**
Foc TR4 F/R^e^	**+++**	**+++**	**+++**	**(+)(+)(+)**

References

^a ^This study

^b ^[29]

^c^ [8]

^d^ [13]

^e^ [10].

The low values of intra-assay CVs (between 0.71% and 0.93%) and inter-assay CVs (between 1.07% and 1.71%) supported the repeatability and the reproducibility of the FWB-TR4 test, respectively. Concerning robustness, no statistical differences between the Ct values were observed after changing the reaction volume (18 μL vs 16 μL; W = 152.5, *p* = 0.20, and 18 μL vs 20 μL, W = 127, *p* = 0.05), the gDNA template (2.0 μL vs 1.8 μL; W = 196.5, *p* = 0.94, and 2.0 μL vs 2.2 μL; W = 227, *p* = 0.48), or the hybridization temperature (60°C vs 58°C; W = 111, *p* = 0.97, and 60°C vs 62°C; W = 143, *p* = 0.21). Likewise, no statistical differences were observed when comparing the Master Mix and the Core Kit (t = 0.41, *p* = 0.69). Nevertheless, comparisons between real-time PCR platforms yielded significantly lower mean Ct values for the Corbett Rotor-Gene platform when compared to the LightCycler 480 (t = 2.3, *p* = 0.03). However, no cross-reaction with closely-related but non-target DNA was observed, thus demonstrating that the TR4-F/P/R qPCR test remained highly specific even in less stringent conditions or using different qPCR equipment.

### Tests on plant material

**Fig 2:**
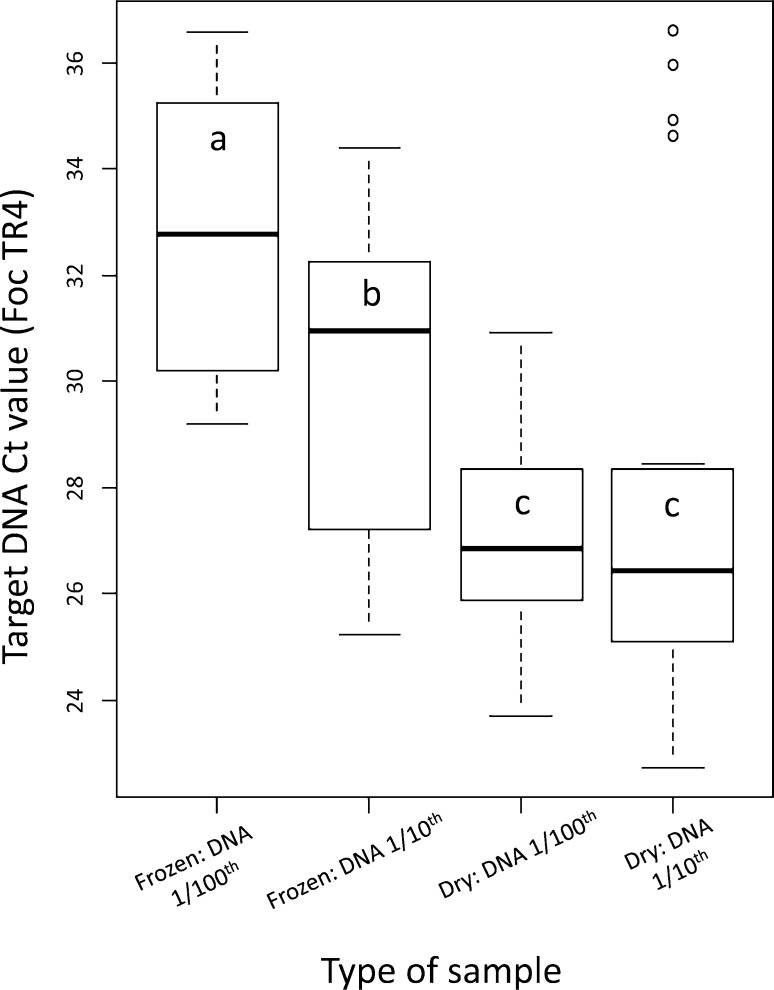
Parametrical comparison between samples pre-treatments before PCR. Comparisons were performed testing the type of sample (dry or frozen banana tissue) and the dilution of the DNA after extraction (raw DNA diluted 10 times or raw DNA diluted 100 times).

The effects of the sample type (dry vs frozen), the DNA dilution (1/10^th^ vs 1/100^th^ dilutions) and their interaction on the mean Ct values were assessed by ANOVA (Table 5). A significant effect was observed for the type of sample, and for the interaction between the type of sample and the DNA dilution. Frozen samples exhibited significantly higher Ct values than dry samples when using post hoc Tukey HSD comparisons between treatments (Fig 2 and Table 6). No statistically significant differences were found between the two dilutions of dry samples, while the frozen samples differed significantly when diluted (Table 6).

**Table 5:** ANOVA table on the effects of dilution (10 times or 100 times), sample type (dry or frozen), and their interaction.

Source of Variation	Df	Sum of squares	Mean square	F	*p* value
Dilution (1/10^th^; 1/100^th^)	1	1.28	1.28	0.16	0.69
Sample type (dry; frozen)	1	443.67	443.67	56.61	<0.001
Dilution * Sample type	1	77.59	77.59	9.90	0.002
Residuals	106	830.78	7.84		

**Table 6:** Post-hoc Tukey Kramer pairwise comparison test between groups of treatments. Samples were either dry or frozen and either diluted 10 times or 100 times.

		Confidence interval		
Type of sample: Type of dilution	Difference	Lower value	Upper value	*p* value
Frozen: DNA 1/10th vs Frozen: DNA 1/100th	-2.76	-4.79	-0.73	0.003
Frozen: DNA 1/100th vs Dry: DNA 1/100th	-5.63	-7.53	-3.72	< 0.0001
Frozen: DNA 1/100th vs Dry: DNA 1/10th	-4.94	-7.14	-2.72	< 0.0001
Frozen: DNA 1/10th vs Dry: DNA 1/100th	-2.87	-4.69	-1.04	0.0004
Frozen: DNA 1/10th vs Dry: DNA 1/10th	-2.18	-4.31	-0.03	0.045
Dry: DNA 1/10th vs Dry: DNA 1/100th	0.69	-1.32	2.71	0.81

Type of dilution: DNA 1/10^th^ (raw DNA diluted 10 times) or DNA 1/100^th^ (raw DNA diluted 100 times).

Difference: Ct mean differences between treatments.

Statistically significant differences were observed when comparing inoculated Foc TR4 dry and frozen samples for 18S Ct (W = 15664; *p* < 0.001) and DNA concentration (W = 6659; *p* < 0.001). Higher mean 18S Ct values were found for dry samples, ranging from 13.1 to 23.5 (mean ± SD; 19.42 ± 2.04). Mean concentration values for dry samples were lower than for frozen samples, ranging from 0.048 to 6.3 ng μL^-1^ (mean ± SD; 1.08 ± 0.96). For frozen samples, 18S Ct values ranged from 7.4 to 21.03 (mean ± SD; 14.71 ± 2.13), while concentration values ranged from 0.043 to 29.70 ng μL^-1^ (mean ± SD; 3.93 ± 4.27). For both types of samples, values for 18S Ct and DNA concentrations out of these boundaries (range of 18S Ct values) gave negative results for Foc TR4, possibly indicating the presence of inhibitory compounds or an excessive dilution of DNA templates.

Seven field samples from Mozambique yielded positive results with the FWB-TR4 test. This was subsequently confirmed by PCR using the Foc TR4 F/R [10] and Foc 0422F2/R2/P2 [29] tests. The samples collected in Trinidad and Tobago, and Reunion tested negative for Foc TR4 (S2 Table).

## Discussion

Foc represents one of the principal threats to banana production worldwide [24], especially since the emergence of strains able to attack Cavendish cultivars. VCG 01213/16, was limited to Southeast Asia and Australia for more than 20 years, but recently emerged in Mozambique, Pakistan and the Middle East [7, 17], indicating that it has moved to new banana-growing countries [4]. The great concern about Foc TR4 is that it might also spread to major banana-producing areas, such as central and eastern Africa, the Indian subcontinent and the Americas [4]. Once the pathogen is established, no control measures are available to protect susceptible cultivars from Fusarium wilt. The implementation of strict quarantine measures is therefore essential to prevent the international movement of Foc TR4 with infected bananas and rhizomes, and to detect Foc TR4 outbreaks early. These measures, however, strongly depend on the availability of a rapid and reliable *in planta* detection tool.

**Fig 3:**
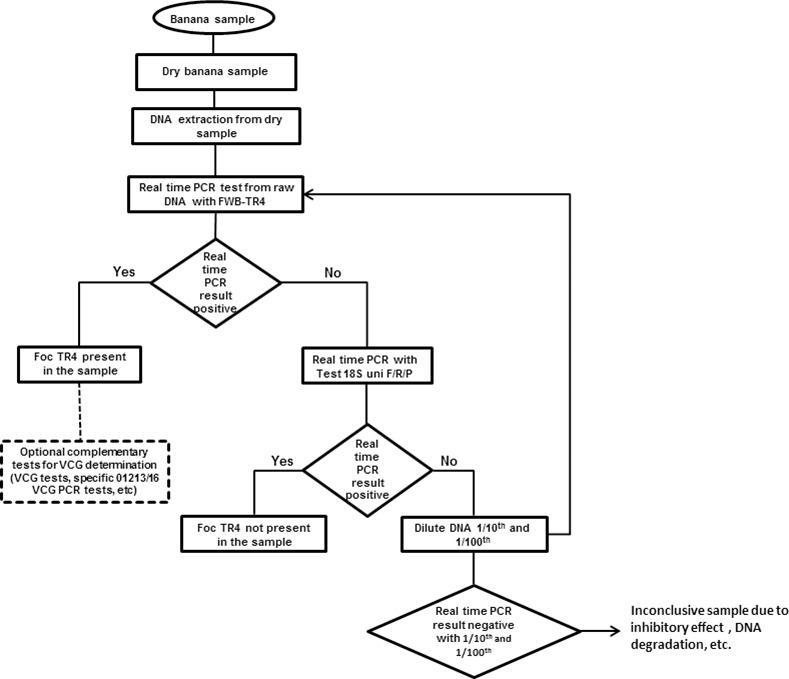
Decision flowchart for PCR detection of tropical strains of Foc race 4 using FWB- TR4 and 18S uni F/R/P primers.

All efforts to develop markers for Foc TR4 have previously focused exclusively on VCG 01213/16, a clone of Foc that is highly aggressive to Cavendish bananas. However, another Foc clone that is phylogenetically closely related to VCG 01213/16, known as VCG 0121, also affects Cavendish bananas in the tropics [14, 19, 55–57]. The current status of VCG 0121 is still under debate and some studies suggest that these strains are part of Foc TR4 [1]. Although VCG 0121 is still restricted to Southeast Asia [19, 55, 56], its introduction into new tropical Cavendish-growing banana regions could potentially have a considerable economic impact. It is thus important that molecular markers developed for Foc TR4 do not detect VCG 01213/16 only, but all forms of Foc able to cause disease to Cavendish bananas in the tropics, including VCG 0121. The criteria used to delimit and identify species, as applied to pathogenic fungi, have changed over time and must take into account morphology, physiology, intersterility, host specificity and phylogenetics [39, 58]. Quarantine regulations based on a list of pathogens that already cause significant disease problems in other parts of the world may lead us to overlook emerging pests, because they do not consider genetic diversity in fungal populations [39]. We think that the movement of new pathogens, and as a consequence, of new alleles should be considered as a whole, taking into account risks posed by invasion genes, transposons or chromosomes into potential pathogens [39]. The close homology of the putative virulence gene described by Li et al. [8] in VCGs 01213/16 and 0121, and the observed differences with other Foc races, provide evidence of the relationship between these two Foc VCGs. In this context, virulence genes, like the one described by Li et al. [8], are considered as ideal targets for the molecular detection of *F*. *oxysporum* strains from different crops and cultivars, which are morphologically indistinguishable [38], as they determinate the host specificity [1, 42]. For these reasons, the detection tool developed in this study targets both VCG 01213/16 and VCG 0121. However, in cases where specific identification of the strains is requested, we recommend further analysis by testing VCG compatibility when the strain is isolated, or by VCG 01213/16-specific tests like W2987 F/R [8], VCG01213/16 F1/R2 [13] and Foc TR4 F/R [10] (Fig 3).

An original approach, using orthogonal arrays (OAs) and the Taguchi method, was employed to improve the sensitivity of this new test without compromising its specificity, using the mean Ct value as the variable. In PCR optimization, OAs may replace the “one-factor-at-a-time” method, in which one factor is varied while the others are kept at predetermined levels [59]. This kind of approach is usually time-consuming and often overlooks the interaction among variables [60]. Combining both methods, we were able to assess the effect of four PCR factors or parameters (concentration of MgCl_2_, primers and probe, the annealing temperature, and the denaturation/polymerization pattern) and their interaction in 18 PCR runs, instead of 64 PCR runs, for a complete factorial design. In biology and biotechnology, OAs and the Taguchi method have been used as a development tool in areas such as microbial fermentations, medicine, molecular biology, food processing and bioremediation [60], including PCR optimization [59, 61–64].

Optimizing the sensitivity of a test is only one of the required steps to propose a robust and validated method. Basic performance criteria should also be assessed, for instance according to the guidelines proposed by EPPO [25, 53, 65, 66], including analytical specificity, sensitivity, repeatability and reproducibility. In addition, robustness is an interesting complementary criterion. The assessment of these performance values may pinpoint potential drawbacks or biases, which could require adjustments in the protocol parameters.

The FWB-TR4 detection tool developed in this study was tested on a comprehensive collection of *Fusarium* isolates, including target and closely related non-target strains, which is necessary to verify the accuracy of the assay. Several studies have shown that Foc presents wide genetic diversity [1, 3, 9, 14], and that the *F*. *oxysporum* species complex comprises non-pathogenic strains that are morphologically undistinguishable from pathogenic ones [10, 67]. This level of complexity is challenging for the development of a race-specific test. Yet, FWB-TR4 proved to be specific for strains of Foc causing disease to Cavendish bananas in the tropics, i.e. VCGs 01213/16 and 0121. Continuous and frequent verification of the specificity of this marker for Foc TR4 is however needed, as more VCGs or genotypes causing disease to Cavendish bananas in the tropics might be discovered in future. Continuous *in vitro* testing of pathogenicity or genetic characterization of Foc strains will also minimize the risk of obtaining false negative and/or positive results.

Comparisons between tests confirmed that all conventional PCR tests that target Foc TR4 are specific to VCG 01213/16. Under our conditions, the Sybr-Green PCR test developed by Lin et al. [26], cross-reacted with DNA from two isolates of Foc race 1 and one strain of *F*. *oxysporum* f. sp. *canariensis*. A plausible explanation for this is that some isolates may be classified as Foc STR4 under subtropical conditions (where they can affect Cavendish banana), but as Foc race 1 in tropical areas, where they are unable to affect Cavendish banana [10]. The cross-reaction with *F*. *oxysporum* f. sp. *canariensis* is more difficult to explain, as no sequences for this gene and for this species are available in any public database. The Foc4 0422F2/R2/P2 probe-based test, described as a Foc race 4 detection tool by Yang et al. [29], was found to be similar to FWB-TR4 in terms of specificity (both tests target VCGs 01213/16 and 0121) and sensitivity, when compared to the conventional PCR tests. However, limited validation data are available for the Foc4 0422F2/R2/P2 test regarding analytical specificity and inclusivity. Our study assessed these two criteria for FWB-TR4 more in-depth by testing a large set of 136 isolates.

The FWB-TR4 test was shown to be highly repeatable and reproducible, and proved to be robust enough to withstand slight pipetting errors or temperature drift during PCR. It also proved to be transferable between PCR machines and mixes (Core Kits vs Master Mixes), without compromising sensitivity and specificity. More importantly, the tests showed satisfactory results when detecting artificially and naturally infected plant material.

It was shown that the pre-treatment of banana samples before analysis was important. It seems that the sensitivity of the PCR was improved when DNA was extracted from previously dried samples rather than from frozen samples. This may have important implications as target positive Ct values vary significantly when treating dry or frozen samples. Furthermore, our tests showed that sensitivity is not compromised when the test is used with a template containing a background of DNA from dried tissue. This is an important finding in cases where samples must be transferred to other laboratories for identification purposes or result confirmation.

We are aware that tropical strains of Foc may be present in other biological matrices like other banana cultivars, or even soil and water. It has been shown that the type of matrix can affect efficiency of PCR detection and that, whenever possible, DNA extraction should be optimized for each matrix [68]. This is of special concern for VCG 01213/16 which attacks several cultivars of banana and plantain [4]. Our molecular test may also be useful to detect Foc TR4 in soil or in water, for regulatory purposes or for epidemiological studies [15, 32], providing that prior optimization of DNA extraction from these environmental matrices is conducted.

Finally, we suggest the introduction of PCR reaction controls. In this study, we propose to include a primer/probe combination that targets a highly conserved region of the ribosomal DNA, which serves as an internal DNA quality control and limits the risk of false negatives. The quality of the gDNA extracted from plant samples should be systematically assessed in the framework of detection analyses. In our case, we used the 18S uni-F/R/P universal primer/probe test. The 18S uni-F/R/P test can be used as an indication to further test gDNA after a supplementary dilution. In this context, we recommend first testing the raw DNA from banana samples with FWB-TR4. If the sample is positive to Foc TR4, the result can be considered as a true positive. If not, we recommend testing the sample with 18S uni F/R/P. If the sample is positive to the universal markers, the sample can be considered as a true negative. If not, raw DNA should be diluted (i.e. 1/10^th^ and 1/100^th^) (Fig 3). Using an empirical approach, we were able to establish reference boundaries for 18S Ct values and gDNA concentrations which, under our conditions, will be useful to control the quality of the extracted DNA in order to obtain reliable results.

## Supporting information

S1 Table List and characteristics of the strains tested in this study and results following the real-time assay targeting a putative pathogenicity gene of tropical strains of *Fusarium oxysporum* f. sp. *cubense* race 4. (XLSX)

S2 Table List and characteristics of naturally infected material tested in this study and results following the real-time assay targeting a putative pathogenicity gene of tropical strains of *Fusarium oxysporum* f. sp. *cubense* race 4. (XLSX)

S3 Table Average signal-to-noise ratios (*n*) for each of the levels tested for the concentration of MgCl2, forward, reverse and probe, the annealing temperature, and the denaturation/polymerization pattern. Three concentrations of plasmid DNA were used for optimization. Optimal values are shown in bold characters.
(PDF)
