# Supplementary material for: Development of a hydrolysis probe-based real-time assay for the detection of tropical strains of Fusarium oxysporum f. sp. cubense race 4
Source: PLoS One. 2017 Feb 8;12(2):e0171767. doi: 10.1371/journal.pone.0171767 (PMC5298334; doi:10.1371/journal.pone.0171767)
Supplement: S3 Table — Three concentrations of plasmid DNA were used for optimization. Optimal values are shown in bold characters. (PDF) [file pone.0171767.s003.pdf]

S3 Table. Average signal-to-noise ratios (*n*) for each of the levels tested for the concentration of $MgCl_2$, forward, reverse and probe, the annealing temperature, and the denaturation/polymerization pattern. Three concentrations of plasmid DNA were used for optimization. Optimal values are shown in bold characters.

	Concentration (pc μL^{-1})		
	**$2.4x10^1$**	**$2.4x10^2$**	**$2.4x10^3$**
**$MgCl_2$ (mM)**			
**4**	**-29.87**	**-28.80**	**-27.57**
**5**	**-29.87**	-28.83	-27.69
**6**	-29.95	-28.93	-27.68
**Effect significance (p value)**	0.132	0.019	0.029
**Primer F (µM)**			
**0.1**	-29.93	-28.88	-27.67
**0.2**	-29.89	**-28.82**	-27.66
**0.3**	**-29.87**	-28.87	**-27.61**
**Effect significance**	0.401	0.185	0.101
**Primer R (µM)**			
**0.1**	-29.94	-28.86	-27.68
**0.2**	-29.91	-28.89	-27.68
**0.3**	**-29.84**	**-28.81**	**-27.59**
**Effect significance**	0.083	0.334	0.476
**Probe (µM)**			
**0.1**	**-29.74**	**-28.68**	**-27.41**
**0.2**	-29.96	-28.89	-27.73
**0.3**	-29.99	-28.99	-27.83
**Effect significance**	<0.001	<0.001	< 0.001
**Annealing (°C)**			
**60**	-29.95	-28.94	-27.67
**62**	-30.00	-28.92	-27.73
**64**	**-29.74**	**-28.74**	**-27.55**
**Effect significance**	<0.001	<0.001	< 0.001
**D/P (s/s)**			
**10/45**	**-29.82**	**-28.79**	**-27.58**
**15/45**	-29.98	-28.92	-27.75
**15/30**	-29.89	-28.85	-27.61
**Effect significance**	0.002	0.0237	< 0.001
